# Development and validation of a predictive nomogram for the risk of MAFLD in postmenopausal women

**DOI:** 10.3389/fendo.2024.1334924

**Published:** 2024-08-06

**Authors:** Ming Yang, Xingyu Chen, Qiaohui Shen, Zhuang Xiong, Tiejun Liu, Yan Leng, Yue Jiao

**Affiliations:** ^1^ College of Traditional Chinese Medicine, Changchun University of Chinese Medicine, Changchun, China; ^2^ Department of Liver, Spleen and Gastroenterology, First Affiliated Hospital to Changchun University of Chinese Medicine, Changchun, China; ^3^ Department of Intensive Care Unit, Changchun Tongyuan Hospital, Changchun, China

**Keywords:** MAFLD, postmenopausal women, nomogram, predictive model, risk factors

## Abstract

**Background and aim:**

Metabolic-associated fatty liver disease (MAFLD) has gradually become one of the main health concerns regarding liver diseases. Postmenopausal women represent a high-risk group for MAFLD; therefore, it is of great importance to identify and intervene with patients at risk at an early stage. This study established a predictive nomogram model of MAFLD in postmenopausal women and to enhance the clinical utility of the new model, the researchers limited variables to simple clinical and laboratory indicators that are readily obtainable.

**Methods:**

Data of 942 postmenopausal women from January 2023 to October 2023 were retrospectively collected and divided into two groups according to the collection time: the training group (676 cases) and the validation group (226 cases). Significant indicators independently related to MAFLD were identified through univariate logistic regression and stepwise regression, and the MAFLD prediction nomogram was established. The C-index and calibration curve were used to quantify the nomogram performance, and the model was evaluated by measuring the area under the receiver operating characteristic curve (AUC), calibration curve, and decision curve analysis (DCA).

**Results:**

Of 37 variables, 11 predictors were identified, including occupation (worker), body mass index, waist-to-hip ratio, number of abortions, anxiety, hypertension, hyperlipidemia, diabetes, hyperuricemia, and diet (meat and processed meat). The C-index of the training group predicting the related risk factors was 0.827 (95% confidence interval [CI] 0.794–0.860). The C-index of the validation group was 0.787 (95% CI 0.728–0.846). Calibration curves 1 and 2 (BS1000 times) were close to the diagonal, showing a good agreement between the predicted probability and the actual incidence in the two groups. The AUC of the training group was 0.827, the sensitivity was 0.784, and the specificity was 0.735. The AUC of the validation group was 0.787, the sensitivity was 0.674, and the specificity was 0.772. The DCA curve showed that the nomogram had a good net benefit in predicting MAFLD in postmenopausal women.

**Conclusions:**

A predictive nomogram for MAFLD in postmenopausal women was established and verified, which can assist clinicians in evaluating the risk of MAFLD at an early stage.

## 1 Introduction

Nonalcoholic fatty liver disease (NAFLD) is a type of clinical-histopathological condition characterized by fat accumulation in hepatocytes, with or without accompanying inflammation, necrosis, and even fibrosis. Globally, the incidence of NAFLD is increasing rapidly, with significant differences observed between men and women (1). Compared with men of the same age, the prevalence rate of NAFLD in premenopausal women is lower; however, this trend reverses after menopause (2, 3). A cross-sectional study in China, which included 9,360 women, found that the incidence of NAFLD was 5.3% in women under 45 years old, 18.8% in women aged 45–55 years, and increased to 27.8% in women over 55 years old (4). With China’s aging population, an increasing number of women are entering the postmenopausal stage, which significantly elevates the risk of liver-related diseases.

To date, the pathogenesis of NAFLD remains ill-defined. Theories of insulin resistance and a “second strike” are widely accepted. Thus, the diagnosis of NAFLD typically relies on exclusion criteria. The gold-standard method for the diagnosis of NAFLD is liver biopsy (5), but it is difficult to achieve in clinical practice owing to its invasive nature. Furthermore, magnetic resonance spectroscopy (MRS) and computed tomography (CT) are difficult to use as early screening methods for NAFLD, owing to their high cost. Ultrasonography (US) is the most common diagnostic method in clinical practice, but its subjectivity leads to significant differences in the findings made by different operators (6), which reduces its sensitivity for the detection of mild fatty liver (7). In 2020, a consensus statement from international experts proposed renaming the condition to metabolic-associated fatty liver disease (MAFLD) and recommended using a combination of imaging and risk factors as the diagnostic standard (8). This new approach has improved the detection rate of MAFLD and underscored the importance of risk factors in its diagnosis.

MAFLD is not just a progressive liver disease; it also contributes to other systemic diseases, particularly cardiovascular diseases (9), which seriously affect the health of postmenopausal women. Owing to the lack of specific clinical symptoms of MAFLD, it is often diagnosed incidentally during physical examination, but it frequently involves inflammatory changes and even fibrosis in the liver. Therefore, it is important to establish a diagnostic prediction model based on easily collected data, such as physical findings, lifestyle, and medical history, which could be readily used by medical institutions such as clinics and community hospitals for the early screening of populations at high risk for MAFLD. This study aimed to analyze MAFLD-related risk factors in postmenopausal women and establish a predictive nomogram to identify high-risk individuals at an early stage.

## 2 Materials and methods

### 2.1 Patient selection

The data of postmenopausal women in Northeast China from January 2023 to October 2023 were collected retrospectively. The diagnosis of MAFLD was based on the presence of steatosis on color Doppler ultrasound accompanied by any of the following conditions: overweight or obesity, diabetes, and metabolic dysfunction. Metabolic dysfunction must meet at least two of the following conditions: 1) waist circumference ≥80 cm; 2) hypertension; 3) plasma triglyceride levels ≥1.70 mmol/L or receiving specific drug treatment; 4) high-density lipoprotein cholesterol ≤1.3 mmol/L; 5) pre-diabetes; 6) high-sensitivity C-reactive protein >2 mg/L (5). This study was approved by the Ethics Committee of the First Affiliated Hospital to Changchun University of Chinese Medicine. All patients consented to the use of their data for the study. No patient received financial compensation.

The inclusion criteria were as follows: women who have been postmenopausal for more than 1 year. The exclusion criteria were as follows (1): unnatural menopause (hysterectomy, ovariectomy, chemotherapy leading to ovarian failure, etc.) (2); specific liver diseases such as viral hepatitis, Wilson’s disease, autoimmune liver disease and drug-induced liver disease that can lead to abnormal liver function (3); a long-term drinking history, generally more than 5 years, with an average daily alcohol consumption in the past 12 months >20 g, or a significant drinking history within the last 2 weeks with an equivalent alcohol consumption of >80 g/d (4); patients with the following medical history are excluded: significant weight loss due to metabolism, history of malignant tumor, history of glucocorticoid treatment, history of intake of medications that affect insulin secretion and sensitivity; or (5) coexisting important organ diseases or serious mental disorders that prevent cooperation with the investigation.

### 2.2 Data collection

This study adopted the field questionnaire survey method. Based on the Clinical Research Guidelines of Metabolic Fatty Liver Disease of the Asia-Pacific Liver Research Association, the Dietary Guidelines for China Residents (2022), and related literature, risk factors were identified and classified, including the patient’s general information (name, age, occupation, education level, and exercise habits), eating habits (cereals, fruits, vegetables, meat and eggs, milk, and processed meat), metabolic risk factors (history of smoking, sleep status, history of hypertension, history of hyperlipidemia, history of diabetes, and history of hyperuricemia), and physical condition. The food frequency questionnaire (FFQ) method was used to assess eating habits. After rigorous training, investigators interviewed respondents and completed the questionnaires to collect data on the frequency of eating and portion sizes of various foods consumed by the respondents in the past 6 months. The FFQ was designed with reference to the common food classifications in Northeast China, including 10 categories and 28 items, ultimately determining the daily intake of each food category.

### 2.3 Statistical analysis

Data entry was performed using Excel. During model development, 942 women were divided into the training group (676 women from January 2023 to July 2023) and validation group (266 women from August 2023 to October 2023) according to the time of case collection. The training dataset was used to develop the model, and the validation dataset was used for external validation. According to Harrell’s (10) guidelines, the final number of variables used for binary logistic regression should not exceed 10% of the number of cases. In the present study, the prediction group included 227 women with MALFD and 449 women without MAFLD. Therefore, the final number of predictors included should not have exceeded 22. The risk factors were analyzed using univariate logistic regression and stepwise regression to eliminate statistically insignificant variables. The coefficient of variance inflation factor (VIF) was utilized to assess the severity of multicollinearity in the multivariate linear regression model, and significant variables were included in the multivariate logistic regression model to identify predictive factors. A nomogram was constructed to build a predictive model, and the nomogram’s performance was evaluated through the calibration of the consistency index (C-index) and 1000 bootstrap samples to minimize overfitting bias. The receiver operating characteristic curve was plotted to assess the internal validity of the prediction model. C-index and area under the receiver operating characteristic (ROC) curve (AUC) values range from 0.5 to 1.0, with 0.5 indicating a random probability and 1.0 indicating perfect fitting. Generally, a C-index and AUC value above 0.7 suggest that the prediction is reliable. A decision curve analysis (DCA) was conducted to evaluate the utility of the clinical prediction model. A *P* value < 0.05 was considered significant. All analyses were performed using R, version 4.3.0.

## 3 Results

### 3.1 General information on patients and univariate logistic regression

**Figure 1:**
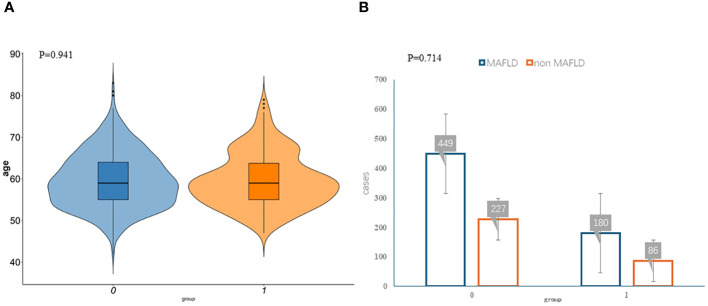
Participant characteristics. The violin plots **(A)** and bar plots **(B)** suggest no significant differences between the two data sets. Group:0 means training group,1 means validation group.

The clinicopathologic variables in this study are reported in **Table 1**. During the study period, we enrolled 942 eligible postmenopausal women with an average age of 59.68 years. According to the diagnostic criteria, 313 women were diagnosed with MAFLD, and the incidence rate was 33.23%. The training group had 676 women, with an average age of 59.66 years, and 227 women with MAFLD (33.58%). The validation group had 266 women, with an average age of 59.70 years, and 86 women had MAFLD (32.33%). No significant differences were observed between the two datasets with regard to baseline characteristics (*P* > 0.05; **Figures 1A, B**). Univariate analysis was used to analyze the risk factors related to fatty liver in postmenopausal women in the training group. Using *P* < 0.1 as the threshold, 20 variables were selected, including age, occupation (workers, housework, employees, and farmers), waist-to-hip ratio, waist-to-height ratio, years since menopause, number of abortions, weight change before and after menopause, body mass index (BMI), hypertension, hyperlipidemia, diabetes, hyperuricemia, Pittsburgh Sleep Quality Index, anxiety, and diet (fruit, meat, and processed meat).

**Table 1:** Baseline characteristics of participants.

Variable	Without MAFLD (n=629)	MAFLD (n=313)	P value
Age (years), mean (SD)/n(%)	59.30 (6.32)	60.42 (6.98)	<0.05
≤50	36 (5.72)	22 (7.03)	
51–60	354 (56.28)	145 (46.33)	
61–70	211 (33.55)	120 (38.34)	
>70	28 (4.45)	26 (8.31)	
Occupation, n (%)			<0.05
Worker	79 (12.56)	66 (21.09)	<0.001
Housewife	127 (20.19)	50 (15.97)	0.119
Office clerk	201 (31.96)	105 (33.55)	0.623
Peasantry	86 (13.67)	27 (8.63)	<0.05
Other	136 (21.62)	65 (20.77)	0.763
Education, n (%)			0.681
Junior high school and below	275 (43.72)	139 (44.41)	
High school	222 (35.29)	113 (36.10)	
University degree or above	132 (20.99)	61 (19.49)	
Exercise, n (%)			0.349
No exercise habit or medium-intensity aerobic exercise for less than 30 minutes per week	584 (92.85)	283 (90.42)	
Moderate intensity aerobic activity ranging from 30 to 150 minutes per week	37 (5.88)	27 (8.63)	
Moderate intensity aerobic exercise for more than 150 minutes per week	8 (1.27)	3 (0.96)	
Weight (kg), mean (SD)	57.31 (7.42)	63.19 (7.90)	<0.001
Height (m), mean (SD)	1.60 (0.044)	1.60 (0.040)	0.385
Waist (cm), mean (SD)	82.40 (8.66)	90.08 (10.34)	<0.001
Hipline (cm), mean (SD)	93.25 (7.95)	96.97 (9.93)	<0.001
Waist-to-hip ratio, median (P25,P75)	0.9 (0.8,0.9)	0.9 (0.9,1.0)	<0.001
Waist-to-height ratio, median (P25,P75)	0.5 (0.5,0.6)	0.5 (0.5,0.6)	<0.001
Age at menopause(years), mean (SD)	50.05 (3.12)	50.48 (3.29)	0.051
Time from menopause(years), median (P25,P75)	8 (4,13)	9 (4,15)	<0.05
Gestational time, median (P25,P75)	2 (1,2)	2 (1,2)	0.191
Abortion times, median (P25,P75), y	0 (0,1)	0 (0,1)	0.1
Premenopausal body weight (kg), mean (SD)	56.81 (6.71)	61.12 (7.52)	<0.001
Premenopausal weight/current weight, median (P25,P75)	1.0 (0.9,1.0)	1.0 (0.9,1.0)	<0.001
Body mass index (kg/m^2^), n (%)	22.35 (2.63)	24.59 (2.94)	<0.001
<23	393 (62.48)	89 (28.43)	
≥23	236 (37.52)	224 (71.57)	
Smoking			0.376
No	615 (97.77)	303 (96.81)	
Yes	14 (2.23)	10 (3.19)	
Hypertension, n (%)			<0.001
No	577 (91.73)	229 (73.16)	
Level 1	39 (6.20)	60 (19.17)	
Level 2	10 (1.59)	15 (4.79)	
Level 3	3 (0.48)	9 (2.88)	
Hyperlipidemia, n (%)			<0.001
No	553 (87.92)	209 (66.77)	
Yes	76 (12.08)	104 (33.23)	
Diabetes, n (%)			<0.001
No	591 (93.96)	254 (81.15)	
Yes	38 (6.04)	59 (18.85)	
Hyperuricemia, n (%)			<0.05
No	623 (99.05)	304 (97.12)	
Yes	6 (0.95)	9 (2.88)	
Pittsburgh Sleep Quality Index, n (%)			0.209
I	213 (33.86)	109 (34.82)	
II	324 (51.51)	171 (54.63)	
III	77 (12.24)	29 (9.27)	
IV	15 (2.38)	4 (1.28)	
Hamilton Anxiety Scale (HAM-A), n (%)			<0.001
no	249 (39.59)	170 (54.31)	
I	294 (46.74)	110 (35.14)	
II	68 (10.81)	26 (8.31)	
III	12 (1.91)	6 (1.92)	
IV	6 (0.95)	1 (0.32)	
Hamilton Depression Scale (HAM-D), n (%)			0.527
no	449 (71.38)	228 (72.84)	
I	163 (25.91)	80 (25.56)	
II	17 (2.70)	4 (1.28)	
III	0 (0)	1 (0.32)	
Staple food intake (g/d), n (%)			0.74
<200	79 (12.56)	45 (14.38)	
200–300	294 (46.74)	130 (41.53)	
>300	256 (40.70)	138 (44.09)	
Vegetable intake (g/d), n (%)			0.327
<300	550 (87.44)	265 (84.66)	
300–500	75 (11.92)	47 (15.02)	
>500	4 (0.64)	1 (0.32)	
Fruit intake (g/d), n (%)			0.605
<200	464 (73.77)	236 (75.40)	
200–350	161 (25.60)	75 (23.96)	
>350	4 (0.64)	2 (0.64)	
Meat intake (g/d), n (%)			<0.001
<120	237 (37.68)	166 (53.04)	
120–200	153 (24.32)	75 (23.96)	
>200	239 (38.00)	72 (23.00)	
Milk intake (mL/d), n (%)			0.81
<300	580 (92.21)	290 (92.65)	
≥300	49 (7.79)	23 (7.35)	
Drinks intake (mL/d), n (%)			0.922
<125	618 (98.25)	309 (98.72)	
125–250	5 (0.79)	0 (0)	
>250	6 (0.95)	4 (1.28)	
Processed meat intake, n (%)			0.45
No	544 (86.49)	265 (84.66)	
Yes	85 (13.51)	48 (15.34)	

BMI = weight (kg)/height (m)^2^, with two decimal places reserved; Waist-to-hip ratio = waist circumference (cm)/hip circumference (cm), with one decimal place reserved; Waist-to-height ratio = waist (cm)/height (cm), with one decimal place reserved; Weight ratio before and after menopause = premenopausal weight (kg)/weight (kg), with one decimal place reserved; Menopausal years = age-menopausal age, with one decimal place reserved; Pittsburgh Sleep Quality index: I, 0–5 points indicate good sleep quality; II, 6–10 points indicate general sleep quality, III, 11–15 points indicate average sleep quality; and IV, 16–21 points indicate poor sleep quality; Hamilton Anxiety Scale (HAM-A): No, <7, I, 7–13 points indicate anxiety; II, 14–20 points indicate anxiety; III, 21–28 points indicate obvious anxiety, and IV≥29 indicates serious anxiety; Hamilton Depression Scale (HAM-D): No < 8 indicates no depressive symptoms, I 8–19 indicate possible depression, II 20–34 points indicate definite depression, and III ≥35 points indicate severe depression.

### 3.2 Selection of predictors of fatty liver in postmenopausal women

The multicollinearity test revealed no statistically significant correlation among the variables. Stepwise regression analysis was performed on the 20 variables, excluding age, occupation (staff), waist-to-height ratio, Pittsburgh Sleep Quality Index, years since menopause, weight change before and after menopause, and fruit consumption frequency. After processing of dummy variables, “housework” and “farmers” within occupations were statistically significant, but not meaningful to the predictive model (odds ratio [OR] < 1; **Table 2**). After excluding the aforementioned variables, 11 key predictors of postmenopausal MAFLD were identified and included in the multivariate logistic regression to construct the model. These predictors are occupation (workers), BMI, waist-to-hip ratio, number of abortions, anxiety, hypertension, hyperlipidemia, diabetes, hyperuricemia, and diet (meat and processed meat). **Table 3** presents detailed information on the predictors.

**Table 2:** Stepwise regression and multicollinearity detection.

Factor added	Deviance	AIC	OR	VIF
None	619.78	647.78		
- Abortion times	621.95	647.95	1.22	1.104
+ Age	618.42	648.42	1.02	1.071
+ Office clerk	618.80	648.80	1.36	1.831
+Waist-to-height ratio	619.02	649.02	5.35	1.679
+ Pittsburgh Sleep Quality Index	619.39	649.39	1.12	1.232
- Hyperuricemia	623.45	649.45	3.82	1.030
+Premenopausal weight/current weight	619.61	649.61	0.61	1.175
+ Fruit intake	619.61	649.61	0.92	1.313
- Worker	624.03	650.03	2.05	1.670
- Hypertension	626.23	652.23	1.60	1.182
- Waist-to-hip ratio	627.10	653.10	9.34	1.547
- Meat intake	627.45	653.45	0.74	1.424
- HAM-A	627.75	653.75	0.67	1.224
- Processed meat intake	629.28	655.28	2.30	1.074
- Housewife	629.85	655.85	0.46	1.579
- Peasantry	630.36	656.36	0.36	1.381
- Diabetes	638.60	664.60	3.97	1.102
- Hyperlipidemia	648.08	674.08	3.57	1.127
- BMI	666.74	692.74	3.96	1.470

**Table 3:** Multivariate logistic regression analysis.

Variable	B	SE	Wald X²	P	OR
Worker	0.8596	0.2577	11.1289	0.000849	2.3622
BMI	1.4504	0.2136	46.1177	1.12E-11	4.2647
Waist-to-hip ratio	3.0815	1.1708	6.9274	0.008489	21.7902
Abortion times	0.2735	0.1333	4.2066	0.040234	1.3146
HAM-A	−0.3471	0.1312	7.0013	0.008151	0.7067
Hypertension	0.4403	0.1958	5.0580	0.024517	1.5532
Hyperlipidemia	1.2775	0.2433	27.5625	1.52E-07	3.5877
Diabetes	1.3201	0.3256	16.4349	5.03E-05	3.7440
Hyperuricemia	1.5524	0.7517	4.2642	0.038907	4.7227
Meat intake	−0.3061	0.1246	6.0319	0.014043	0.7363
Processed meat intake	0.8850	0.2781	10.1251	0.001461	2.4229

### 3.3 Establishment and evaluation of the predictive nomogram model for fatty liver in postmenopausal women

**Figure 2:**
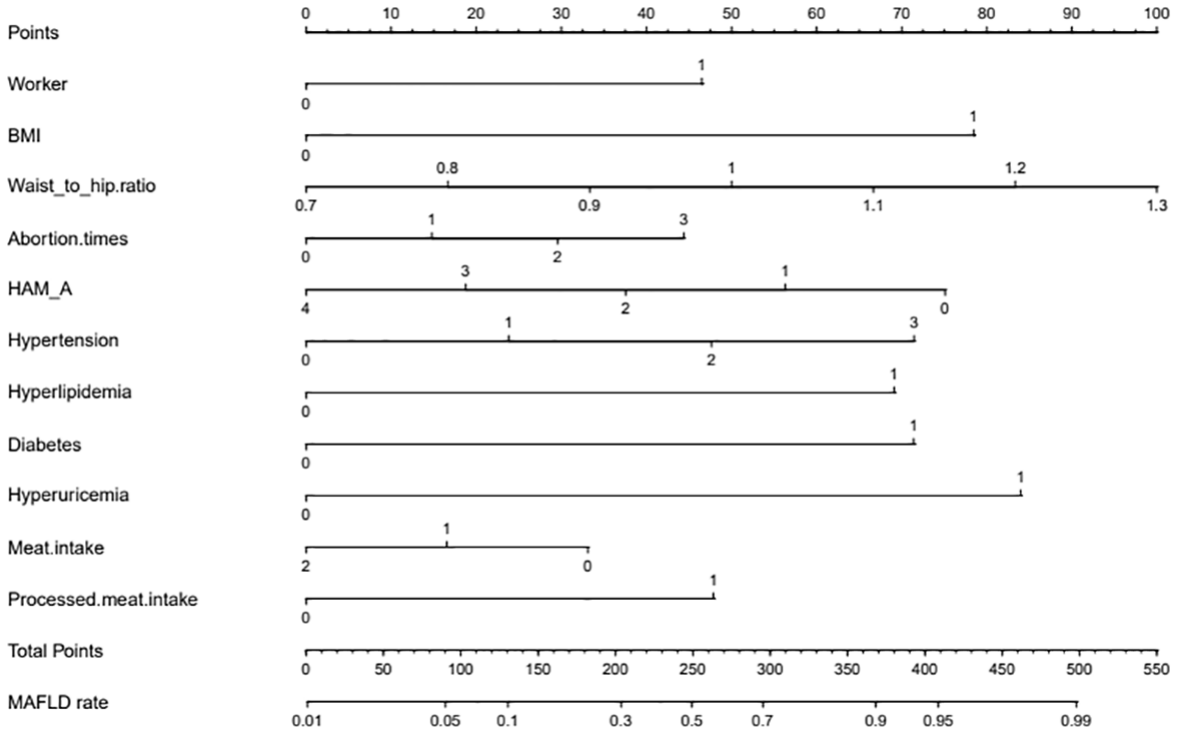
Nomogram. The nomogram represents the prediction probability of MAFLD, ranging from 0 to 550. For each predictive, a vertical line is drawn to the point axis, and the corresponding point is noted down. The scores of each predictor are summed. The total score corresponding to the predicted occurrence probative variability of MAFLD is provided at the bottom of the nomogram. Worker: 0 means non-worker; 1 means workers. BMI: 0 means less than 23 kg/m^2^, and 1 means greater than or equal to 23 kg/m^2^; HAM-A: 0 means no anxiety, 1 means I-degree anxiety, 2 means II-degree anxiety, 3 means III-degree anxiety, and 4 means IV-degree anxiety; Hypertension: 0 means no hypertension, 1 means hypertension grade I, 2 means hypertension grade II, and 3 means hypertension grade III; Hyperlipidemia:0 means no hyperlipidemia, and 1 means hyperlipidemia; Diabetes: 0 means no diabetes, 1 means diabetes; Hyperuricemia: 0 means no hyperuricemia, 1 means hyperlipidemia; Meat intake: 0 means that the average daily meat intake is less than <120 g, 1 means that the average daily meat intake is 120–200 g, and 2 means that the average daily meat intake is more than 200 g; Processed meat intake: 0 means that the diet does not contain processed meat, and 1 means that the diet contains processed meat.

**Figure 3:**
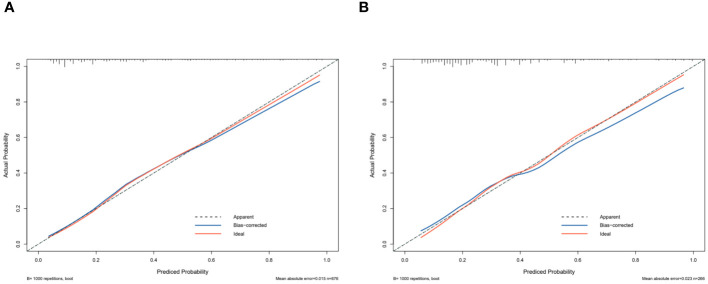
Receiver operating characteristic curve. The performance of the new nomogram was assessed by calibration curves in the training dataset **(A)** and the validation dataset **(B)**. The X-axis represents specificity; the Y-axis represents sensitivity.

**Figure 4:**
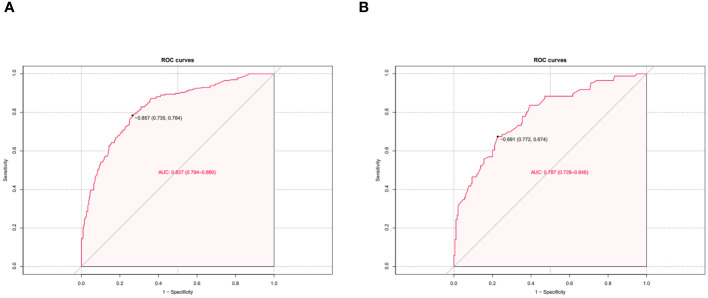
Predictive model calibration curve. ROC curves for predicting MAFLD in the training dataset **(A)** and the validation dataset **(B)**. The X-axis is the predictive probability of the nomogram for MAFLD; the Y-axis represents the actual probability.

**Figure 5:**
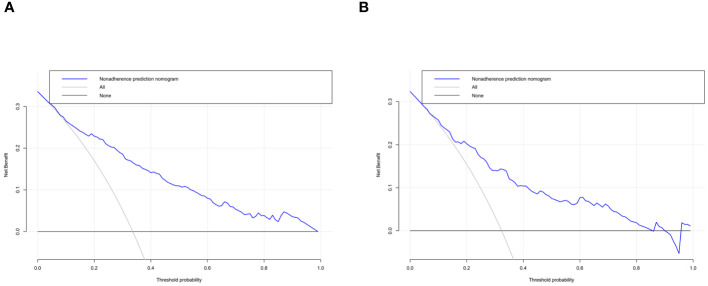
Decision curve analysis. The clinical utility of the nomogram was evaluated by decision curves in the training dataset **(A)** and the validation dataset **(B)**. The X-axis measures the threshold probability. The Y-axis represents net benefits, calculated by subtracting the relative harms (false positives) from the benefits (true positives).

The independent risk factor data obtained from the multivariate logistic regression analysis were input into R software to construct the nomogram risk prediction model (**Figure 2**). The C-index for the prediction group was 0.827 (95% CI 0.794 to 0.860) and that for the verification group was 0.787 (95% CI 0.728 to 0.846), indicating moderate accuracy. BS 1,000 times was used to verify the accuracy of the predicted model. The training group BS 1,000 times drew calibration curves (**Figure 3A**), and the model curve and the actual curve were close to the diagonal line, with an absolute error of 0.015. For the verification group, BS 1,000 times was also used to draw calibration curves (**Figure 3B**), and the model curve and the actual curve were also close to the diagonal line, with an absolute error of 0.023. Thus, the results show that the predicted probability of the model agreed well with the actual incidence in both groups. The AUC represents the area under the ROC curve, serving as an evaluation metric capable of quantifying and categorizing model performance. As the AUC value approaches 1, it signifies higher accuracy levels within the model. The AUC value for the prediction group was 0.827, the sensitivity was 0.784, and the specificity was 0.735 (**Figure 4A**). The AUC value of the verification group was 0.787, the sensitivity was 0.674, and the specificity was 0.772 (**Figure 4B**). The DCA curve showed that the nomogram had a good net benefit in predicting MAFLD in postmenopausal women (**Figures 5A, B**).

## 4 Discussion

The core feature of MAFLD is hepatic steatosis (8). However, liver biopsy, MRI, CT scans, and other diagnostic tests are not suitable for early mass screening, owing to the limitations described above. Previous studies have demonstrated that scoring systems can serve as an early and straightforward evaluation method for MAFLD. Current MAFLD scores primarily rely on anthropometric parameters in combination with non-invasive biochemical parameters, such as the hepatic steatosis index (HSI), the fatty liver index (FLI), and the visceral adiposity index (VAI) (11). These scores exhibit good levels of accuracy for their respective populations. Nevertheless, there has been a lack of research regarding appropriate models of MAFLD for use in postmenopausal women, and the existing score models require biochemical measurements that may not be suitable for early screening in lower-tier hospitals. In the present study, we collected easily obtainable demographic data (age, occupation, etc.), anthropometric data (waist-to-hip ratio, BMI, etc.), details of lifestyle (dietary patterns, exercise routines), and information regarding comorbidities (hypertension, diabetes, etc.) from postmenopausal women attending hospitals or physical examination centers of all types. The resulting nomogram represents a simple, yet practical, diagnostic tool. For each predictive, a vertical line is drawn to the point axis, and the corresponding point is noted down. The scores of each predictor are summed. The total score corresponding to the predicted occurrence probative variability of MAFLD is provided at the bottom of the nomogram. According to the occurrence and progression of diseases as well as the characteristics of health factors, preventive medicine classifies prevention strategies into three levels: primary prevention (etiological prevention), secondary prevention (pre-clinical prevention), and tertiary prevention (clinical prevention). Our model can assist physicians in assessing patients without relying on laboratory tests or imaging evidence, providing recommendations on whether further examinations are necessary based on the results. It is specifically designed for community-level secondary prevention of postmenopausal MAFLD, aiming to achieve early detection, diagnosis, and treatment with the goal of reversing, halting, or delaying the progression of fatty liver disease. Additionally, doctors can utilize the outcomes from this nomogram to deliver health education regarding lifestyle choices, diet modifications, and exercise routines for postmenopausal women in order to accomplish primary preventive measures.

In this study, the detection rate of fatty liver in postmenopausal women was 33.23%. The prevalence of MAFLD is higher than that in the general population (12, 13). In this study, 11 predictors from 37 variables were identified, and they were used to construct a risk nomogram for diagnosing postmenopausal MAFLD. The validation of the nomogram demonstrates that it has good predictive and calibration abilities. Notably, hypertension, hyperlipidemia, hyperuricemia, and diabetes are typical metabolic disorders that also serve as risk factors for the diagnosis of MAFLD in non-postmenopausal women (5).

BMI and waist-to-hip ratio are also primary predictors in the nomogram. The relationship between obesity and metabolic diseases has been widely discussed. In a cohort study of 23,993 cases, Ofer et al. discovered that a BMI of below 27 kg/m^2^ offered an ideal negative predictive value for metabolic syndrome (MetS) (14). BMI is also indicative of long-term health outcomes, and adolescent obesity is an important risk factor for adult MetS (15, 16). Ying et al. conducted a 16-year follow-up study on 554 adolescents and discovered that those with rapidly increasing BMI had a significantly higher risk of MetS than those with slower BMI growth (17). Our study found that the ratio of premenopausal weight to current weight was correlated with postmenopausal MAFLD in univariate logistic regression. Obesity is closely linked to other metabolic and endocrine-related organ diseases. Milewska et al. observed 105 patients with a BMI ≥ 30 kg/m^2^ and further classified them into classes I, II, and III obesity, finding that the prevalence of hyperlipidemia, hypertension, asthma, and obstructive sleep apnea increased with the level of obesity (18). In our study, BMI was also identified as a risk factor for the development of MAFLD in postmenopausal women (OR = 4.2647). However, women’s body fat tends to be more concentrated in the buttocks and thighs than in the upper body before menopause (19). The decrease in estrogen and sex hormone-binding globulin levels, along with increases in follicle-stimulating hormone, androgens, and the androgen/estrogen ratio, may affect the development of fat cells in specific areas, leading to higher abdominal obesity in postmenopausal women compared to premenopausal women (20). Additionally, as age increases, the loss of muscle tissue and the gain in fat content typically affect the accuracy of BMI as a predictive measure. Therefore, it is essential to consider the influence of body fat distribution on metabolism-related diseases in postmenopausal women. Kim et al. found that an increase in visceral adipose tissue area was positively correlated with the incidence of NAFLD, while an increase in subcutaneous adipose tissue area was significantly correlated with the reversal of NAFLD (21). Hong et al. discovered that BMI, waist circumference, and waist-to-hip ratio have a similar potential in predicting the risk of NAFLD in premenopausal women, with the waist-to-hip ratio being the most significant risk factor among postmenopausal women (22). This aligns with our model, which suggests that the waist-to-hip ratio is a more important predictor of MAFLD in postmenopausal women (OR = 21.7902), indicating that accumulation of abdominal fat in postmenopausal women is more likely to lead to MAFLD than an increase in BMI alone.

Although diet is one of the causes of MAFLD, the specific role of dietary factors in the occurrence and development of the disease remains unclear. A cross-sectional study conducted in the United States, involving 3,573 subjects, adopted five dietary quality standards, including the Dietary Inflammation Index (DII), the Mediterranean diet, the Dietary Approaches to Stop Hypertension, the Alternative Healthy Eating Index, and the Healthy Eating Index, to observe their relationship with MAFLD. It was found that the DII was positively correlated with MAFLD, while the other four were negatively correlated (23). However, the correlation between the intake of specific food components and MAFLD has not been well studied. Referring to the Dietary Guidelines for Chinese Residents (2022), this study classified foods and graded their intake. According to our statistics, there was a negative correlation between meat consumption and the occurrence of MAFLD (OR=0.7363). The effect of meat on diseases has always been controversial. Studies in the United States and Europe typically categorize meat as red or white. For instance, research by the National Institutes of Health suggested that red meat and saturated fat may be associated with an increased risk of chronic liver disease and liver cancer (hepatocellular carcinoma [HCC]), while white meat might reduce this risk (24). In 2015, consumption of red meat was classified as “possibly carcinogenic to humans” (25). However, subsequent studies have also provided evidence that red meat does not necessarily cause cancer. Ma (26) and others have found that the intake of processed meat may be positively related to the risk of HCC, while fish and poultry may decrease the risk. When designing our questionnaire, this study also considered the differences between fresh and processed meats. Because of Western dietary habits, most studies do not differentiate between fresh and processed red meats, which may contribute to the controversy over the impact of red meat on disease. However, given China’s preference for fresh foods, processed meat is not commonly consumed by the older population. Therefore, in our questionnaire, we separately accounted for the consumption of processed meats and found that postmenopausal women who consume processed meats are more likely to develop MAFLD (OR=2.4229), which was included as an independent risk factor in the predictive model. Lipoprotein metabolism in the liver, which encompasses cholesterol metabolism, triglyceride metabolism, lipoprotein synthesis, and secretion, is a vital part of lipid metabolism. These pathways together regulate the processes of lipid synthesis, transport, and degradation, and an adequate intake of protein is crucial for maintaining these processes. Compared with fresh red meat, processed meat may contain potential carcinogens such as N-nitroso compounds or heterocyclic amines that remain after the cooking process, in addition to high levels of saturated fat and heme iron, all of which are possible pathogenic factors in processed meats. Ideally, the preservation and cooking methods of meat should be further considered, and our findings require more research for verification.

Considering the relationship between emotional disorders and MetS, it is reasonable to posit a correlation between mental health status and MAFLD. Kim et al. analyzed 10,484 samples in the United States and found that subjects with depression had a higher risk of NAFLD, suggesting depression may be an independent risk factor for NAFLD (27). Anxiety and depression can also affect the prognosis of MAFLD. Tomeno et al. observed the therapeutic effects on 258 patients with major depressive disorder by adjusting their lifestyles, including 32 patients with NAFLD and reported that these patients had a poorer therapeutic response. This may be attributable to the influence of emotional factors on memory and self-efficacy, resulting in poor compliance (28). However, some studies have reported different results. In the systematic review by Tang (29), two cohort studies were included, which reported no correlation between MetS and anxiety (30, 31). Akbari conducted a cross-sectional analysis as part of a prospective cohort study in Isfahan and discovered that anxiety was negatively correlated with MetS, but found no correlation between depression and MetS (32). This finding aligns with those obtained in the present study, which also revealed that anxiety is negatively correlated with MAFLD in postmenopausal women (OR=0.7067). It is commonly believed that the inflammatory response triggered by emotional stress is a contributing factor to MAFLD, yet anxiety and depression also affect eating habits. This influence is often bidirectional, which may explain the contradictory effects on MAFLD.

## 5 Limitation

While the use of estrogen to prevent and treat fatty liver in postmenopausal women is controversial (6), the correlation between estrogen and postmenopausal MAFLD is certain. Klair et al. (33) analyzed the menopause timing of 488 postmenopausal women and the duration since menopause at the time of their examination, finding that prolonged estrogen deficiency in a postmenopausal state increased the risk of liver fibrosis in women with NAFLD. Lu et al. (34) investigated 4,128 postmenopausal women and found that those with earlier menarche had a higher risk of overweight/obesity, insulin resistance, and NAFLD. Menstrual history is also associated with other metabolic diseases. Chen et al. (35) observed a significant correlation between menstrual history, serum uric acid levels, and NAFLD, positing that this may be related to decreased estrogen levels. In univariate logistic regression, the time since menopause and the number of abortions were associated with postmenopausal MAFLD; after adjustment, only the number of abortions remained significant. However, the duration of menstruation, menstrual cycle regularity, and the number of pregnancies were not included in the predictive model in this study. The use of a retrospective study to analyze menstrual history may have some limitations, and this study designed prospective studies with serological markers to longitudinally track this issue.

Exercise has always been a focal point in the prevention and treatment of metabolic diseases. A position statement from Exercise and Sport Science Australia suggests that moderate-intensity aerobic exercise for at least 150–240 minutes per week can reduce liver steatosis by 2%–4%, and high-intensity interval training (HIIT) may also have a similar effect (36). In the present research, this correlation was not observed, which may be attributable to the fact that postmenopausal women in China, generally being of retirement age and primarily engaged in housework, lack sustained exercise of moderate intensity or higher. Nevertheless, multivariate regression analysis revealed that occupation (being a worker) is an independent risk factor for postmenopausal MAFLD, potentially related to the increase in body fat due to a sudden decrease in physical activity after retirement. Additionally, despite rigorous training of investigators, it remains challenging for respondents to have a uniform understanding of what constitutes moderate-intensity exercise, and we cannot retroactively determine objective indicators such as respondents’ heart rates during exercise. These factors may contribute to the reasons why exercise was not included as a risk factor in the final analysis.

## 6 Conclusion

In this study, an effective clinical nomogram was developed for a specific group—postmenopausal women. The model is based on 11 clinically accessible and objective variables, which can be utilized for the early screening of MAFLD in postmenopausal women. For those identified as high risk for MAFLD, laboratory examinations should be enhanced to establish individualized treatment plans at an early stage. Therefore, this model holds significant clinical value. However, further validation is necessary to confirm its widespread applicability.

## Data availability statement

The original contributions presented in the study are included in the article/**Supplementary Material**. Further inquiries can be directed to the corresponding authors.

## Ethics statement

The studies involving humans were approved by The Ethics Committee of the First Affiliated Hospital to Changchun University of Chinese Medicine. The studies were conducted in accordance with the local legislation and institutional requirements. Written informed consent for participation was not required from the participants or the participants’ legal guardians/next of kin in accordance with the national legislation and institutional requirements.

## Author contributions

MY: Writing – review & editing, Writing – original draft, Validation, Methodology, Investigation, Data curation, Conceptualization. XC: Writing – review & editing, Data curation. QS: Writing – review & editing, Investigation, Data curation. ZX: Writing – review & editing, Methodology, Data curation. TL: Writing – review & editing, Methodology, Investigation. YL: Writing – review & editing. YJ: Writing – review & editing, Data curation.
