# Supplementary material for: Development and validation of a predictive nomogram for the risk of MAFLD in postmenopausal women
Source: Front Endocrinol (Lausanne). 2024 Aug 6;15:1334924. doi: 10.3389/fendo.2024.1334924 (PMC11334217; doi:10.3389/fendo.2024.1334924)
Supplement: Supplementary file 2 [file Table_1.docx]

Attached table: Characteristics of Participants in Training Group and Verification Group

|  | Training group(n=676) | | Validation group(n=266) | |
| --- | --- | --- | --- | --- |
| Variable | Without MAFLD(n=449) | MAFLD(n=227) | Without MAFLD(n=180) | MAFLD(n=86) |
| Age(years), mean (SD) /n(%) | 59.05(6.35) | 60.89(6.96) | 59.94(6.20) | 59.20(6.94) |
| ≤50 | 29(6.46) | 13(5.73) | 7(3.89) | 9(10.47) |
| 51–60 | 249(55.46) | 99(43.61) | 105(58.33) | 46(53.49) |
| 61–70 | 153(34.08) | 94(41.41) | 58(32.22) | 26(30.23) |
| ＞70 | 18(4.01) | 21(9.25) | 10(5.56) | 5(5.81) |
| Occupation, n(%) |  |  |  |  |
| Worker | 61(13.59) | 52(22.91) | 18(10.00) | 14(16.28) |
| Housewife | 86(19.15) | 28(12.33) | 41(22.78) | 22(25.58) |
| Office clerk | 135(30.07) | 87(38.32) | 66(36.67) | 18(20.93) |
| Peasantry | 71(15.81) | 17(7.49) | 15(8.33) | 10(11.63) |
| Other | 96(21.38) | 43(18.94) | 40(22.22) | 22(25.58) |
| Education, n(%) |  |  |  |  |
| Junior high school and below | 193(42.98) | 90(39.65) | 82(45.56) | 49(56.98) |
| High school | 175(38.98) | 87(38.33) | 47(26.11) | 26(30.23) |
| University degree or above | 81(18.04) | 50(22.03) | 51(28.33) | 11(12.79) |
| Exercise, n(%) |  |  |  |  |
| No exercise habit or medium-intensity aerobic exercise for less than 30 minutes per week | 408(90.87) | 202(88.99) | 176(97.78) | 81(94.19) |
| Moderate intensity aerobic activity ranging from 30 to 150 minutes per week | 34(7.57) | 23(10.13) | 3(1.67) | 4(4.65) |
| Moderate intensity aerobic exercise for more than 150 minutes per week | 7(1.56) | 2(0.88) | 1(0.56) | 1(1.16) |
| Weight (kg), mean (SD) | 57.01(7.65) | 64.30(8.15) | 58.07(6.76) | 60.25(6.33) |
| Height (m), mean (SD), | 1.60(0.04) | 1.61(0.04) | 1.60(0.04) | 1.60(0.04) |
| Waist (cm), mean (SD), | 82.71(8.79) | 89.54(10.62) | 81.63(8.29) | 91.50(9.47) |
| Hipline (cm), mean (SD) | 93.13(7.87) | 97.73(10.13) | 93.53(8.15) | 94.94(9.15) |
| Waist-to-hip ratio, median (P25, P75) | 0.9(0.8,0.9) | 0.9(0.9,1.0) | 0.9(0.9,0.9) | 0.9(0.8,1.0) |
| Waist-to-height ratio, median (P25, P75) | 0.5(0.5,0.5) | 0.6(0.5,0.6) | 0.5(0.5,0.6) | 0.5(0.5,0.6) |
| Age at menopause（years）, mean (SD) | 49.89(3.14) | 50.18(3.47) | 50.44(3.06) | 51.27(2.63) |
| Time from menopause（years）, median (P25, P75) | 8(4,13) | 10(5,15.5) | 7.5(4,13) | 7.5(4.14) |
| Gestational time, median (P25, P75) | 2(1,2) | 2(1,3) | 2(1,2) | 2(1,2) |
| Abortion times, median (P25, P75), y | 0(0,1) | 0(0,1) | 0(0,1) | 0(0,1) |
| Premenopausal body weight, mean (SD) | 56.49(6.82) | 62.21(7.66) | 57.62(6.37) | 58.26(6.31) |
| Premenopausal weight/current weight, median (P25, P75) | 1.0(0.9,1.0) | 1.0(0.9,1.0) | 1.0(0.9,1.0) | 1.0(0.9,1.0) |
| Body mass index, (kg/m2) , mean(SD)/ n(%) | 22.37(2.51) | 24.41(2.94) | 22.54(2.42) | 23.62(2.21) |
| ＜23 | 514(62.99) | 121(30.56) | 109(60.56) | 34(39.53) |
| ≥23 | 302(37.01) | 275(69.44) | 71(39.44) | 52(60.46) |
| Smoking, n(%) |  |  |  |  |
| No | 440(97.99) | 220(96.92) | 175(97.22) | 83(96.51) |
| Yes | 9(2.01) | 7(3.08) | 5(2.78) | 3(3.49) |
| Hypertension, n(%) |  |  |  |  |
| No | 414(92.20) | 254(67.84) | 163(90.56) | 75(87.21) |
| Level 1 | 24(5.35) | 52(22.91) | 15(8.33) | 8(9.30) |
| Level 2 | 9(2.00) | 12(5.29) | 1(0.56) | 3(3.49) |
| Level 3 | 2(0.45) | 9(3.96) | 1(0.56) | 0(0.00) |
| Hyperlipidemia, n(%) |  |  |  |  |
| No | 398(88.64) | 130(57.27) | 155(86.11) | 25(13,89) |
| Yes | 51(11.36) | 97(42.73) | 25(13.89) | 7(8.14) |
| Diabetes, n(%) |  |  |  |  |
| No | 426(94.88) | 178(78.41) | 165(91.67) | 76(88.37) |
| Yes | 23(5.12) | 49(21.59) | 15(8.33) | 10(11.63) |
| Hyperuricemia, n(%) |  |  |  |  |
| No | 446(99.33) | 218(96.04) | 177(98.33) | 86(100.00) |
| Yes | 3(0.67) | 9(3.96) | 3(1.67) | 0(0.00) |
| Pittsburgh Sleep Quality Index, n(%) |  |  |  |  |
| I | 135(30.07) | 84(37.00) | 78(43.33) | 25(29.07) |
| II | 237(52.78) | 114(50.22) | 87(48.33) | 57(60.28) |
| III | 65(14.48) | 25(11.01) | 12(6.67) | 4(4.65) |
| IV | 12(2.67) | 4(1.76) | 3(1.67) | 0(0.00) |
| Hamilton Anxiety Scale (HAM-A), n(%) |  |  |  |  |
| no | 164(36.52) | 114(50.22) | 85(47.22) | 56(65.12) |
| I | 210(46.77) | 81(35.68) | 84(46.67) | 29(33.72) |
| II | 60(13.36） | 25(11.01) | 8(4.44) | 1(1.16) |
| III | 9(2.00) | 6(2.64) | 3(1.67) | 0(0.00) |
| IV | 6(1.34) | 1(0.44) |  |  |
| Hamilton Depression Scale (HAM-D), n(%) |  |  |  |  |
| no | 321(71.49) | 166(73.13) | 128(71.11) | 62(72.09) |
| I | 115(25.61) | 57(25.11) | 48(26.67) | 23(26.74) |
| II | 13(2.90) | 3(1.32) | 4(2.22) | 1(1.16) |
| III | 0(0) | 1(0.44) | 0(0.00) | 0(0.00) |
| Staple food intake (g/d), n(%) |  |  |  |  |
| ＜200 | 69(15.37) | 41(18.06) | 10(5.56) | 4(4.65) |
| 200–300 | 211(46.99) | 111(48.90) | 83(46.11) | 19(48.33) |
| ＞300 | 169(37.64) | 75(33.04) | 87(48.33) | 63(73.26) |
| Vegetable intake (g/d), n(%) |  |  |  |  |
| ＜300 | 390(86.86) | 191(84.14) | 160(88.89) | 74(86.05) |
| 300–500 | 56(12.47) | 35(15.42) | 19(10.56) | 12(13.95) |
| ＞500 | 3(0.67) | 1(0.44) | 1(0.56) | 0(0.00) |
| Fruit intake (g/d), n(%) |  |  |  |  |
| ＜200 | 319(71.05) | 177(77.97) | 145(80.56) | 35(68.60) |
| 200–350 | 126(28.06) | 48(21.15) | 35(19.44) | 27(31.39) |
| ＞350 | 4(0.89) | 2(0.88) | 0(0.00) | 0(0.00) |
| Meat intake (g/d), n(%) |  |  |  |  |
| ＜120 | 151(33.63) | 101(44.49) | 86(47.78) | 65(75.58) |
| 120–200 | 129(28.73) | 67(29.52) | 24(13.33) | 8(9.30) |
| ＞200 | 169(37.64) | 59(25.99) | 70(38.89) | 13(15.11) |
| Milk intake (mL/d), n(%) |  |  |  |  |
| ＜300 | 405(90.20) | 206(90.75) | 175(97.22) | 84(97.67) |
| ≥300 | 44(9.80) | 21(9.25) | 5(2.78) | 2(2.33) |
| Drinks intake (mL/d), n(%) |  |  |  |  |
| ＜125 | 441(98.22) | 223(98.24) | 177(98.33) | 86(100.00) |
| 125–250 | 3(0.67) | 0(0) | 2(1.11) | 0(0.00) |
| ＞250 | 5(1.11) | 4(1.76) | 1(0.56) | 0(0.00) |
| Processed meat intake, n(%) |  |  |  |  |
| No | 399(88.86) | 187(82.38) | 145(80.56) | 78(90.70) |
| Yes | 50(11.14) | 40(17.62) | 35(19.44) | 8(9.30) |

BMI = weight (kg)/height (m)^2^, with two decimal places reserved; Waist-to-hip ratio = waist circumference (cm)/hip circumference (cm), with one decimal place reserved; Waist-to-height ratio = waist (cm)/height (cm), with one decimal place reserved; Weight ratio before and after menopause = premenopausal weight (kg)/weight (kg), with one decimal place reserved; Menopausal years = age-menopausal age, with one decimal place reserved; Pittsburgh Sleep Quality index: I, 0–5 points indicate good sleep quality; II, 6–10 points indicate general sleep quality, III, 11–15 points indicate average sleep quality; and IV, 16–21 points indicate poor sleep quality; Hamilton Anxiety Scale (HAM-A): No, <7, I, 7–13 points indicate anxiety; II, 14–20 points indicate anxiety; III, 21–28 points indicate obvious anxiety, and IV≥29 indicates serious anxiety; Hamilton Depression Scale (HAM-D): No < 8 indicates no depressive symptoms, I 8–19 indicate possible depression, II 20–34 points indicate definite depression, and III ≥35 points indicate severe depression.
